# Aging-associated accumulation of mitochondrial DNA mutations in tumor origin

**DOI:** 10.1093/lifemedi/lnac014

**Published:** 2022-08-17

**Authors:** Minghua Kong, Lishu Guo, Weilin Xu, Chengpeng He, Xiaoyan Jia, Zhiyao Zhao, Zhenglong Gu

**Affiliations:** Division of Nutritional Sciences, Cornell University, Ithaca, NY 14853, USA; School of Life Sciences, Westlake University, Hangzhou 310024, China; Center for Mitochondrial Genetics and Health, Greater Bay Area Institute of Precision Medicine (Guangzhou), Fudan University, Guangzhou 511400, China; Division of Nutritional Sciences, Cornell University, Ithaca, NY 14853, USA; Center for Mitochondrial Genetics and Health, Greater Bay Area Institute of Precision Medicine (Guangzhou), Fudan University, Guangzhou 511400, China; Center for Genomic Technologies, Greater Bay Area Institute of Precision Medicine (Guangzhou), Fudan University, Guangzhou 511400, China; Center for Mitochondrial Genetics and Health, Greater Bay Area Institute of Precision Medicine (Guangzhou), Fudan University, Guangzhou 511400, China; Division of Nutritional Sciences, Cornell University, Ithaca, NY 14853, USA; Center for Mitochondrial Genetics and Health, Greater Bay Area Institute of Precision Medicine (Guangzhou), Fudan University, Guangzhou 511400, China

**Keywords:** aging, mtDNA mutation, metabolism, genome instability, tumorigenesis

## Abstract

The majority of cancer patients are among aged population, suggesting an urgent need to advance our knowledge on complicated relationship between aging and cancer. It has been hypothesized that metabolic changes during aging could act as a driver for tumorigenesis. Given the fact that mitochondrial DNA (mtDNA) mutations are common in both tumors and aged tissues, it is interesting to contemplate possible role of age-related mtDNA mutations in tumorigenesis. MtDNA encodes genes essential for mitochondrial metabolism, and mtDNA mutates at a much higher rate than nuclear genome. Random drifting of somatic mtDNA mutations, as a result of cell division or mitochondrial turnover during aging, may lead to more and more cells harboring high-frequency pathogenic mtDNA mutations, albeit at different loci, in single-cells. Such mutations can induce metabolic reprogramming, nuclear genome instability and immune response, which might increase the likelihood of tumorigenesis. In this review, we summarize current understanding of how mtDNA mutations accumulate with aging and how these mutations could mechanistically contribute to tumor origin. We also discuss potential prevention strategies for mtDNA mutation-induced tumorigenesis, and future works needed in this direction.

## Introduction

Aging is almost a universal feature of multicellular organisms, characterized by progressive loss of functions at cellular, tissue, and organism levels [1]. One of the most prominent hallmarks of aging is compromised energy metabolism. Age-related metabolic and neurodegenerative diseases, such as diabetes, Parkinson’s, and Alzheimer’s diseases, have rapidly increased in prevalence over the past decades [2]. Cancer is also an age-related degenerative disease, and cancer incidence increased exponentially after mid-point of lifespan [3]. Approximately half of cancers are diagnosed in patients aged more than 65 years, and most malignancies are more prevalent in elderly patients [4]. At present, aging is considered to be a crucial risk factor for cancer. The most accepted hypothesis ‘multi-hit’ proposed that aging process provides substantial time to accumulate potential genomic mutations, which can direct cells into a tumorigenesis fate [5]. Indeed, DNA damage-harboring cells were commonly found in aging tissues [6]. However, it has been observed in many independent studies that calorie restriction and exercise intervention can significantly reduce cancer risk [7, 8]. By contrast, the overload of calories and/or a sedentary lifestyle has a reverse effect [9, 10]. Therefore, there might be correlations among age-related genetic alterations, metabolism and carcinogenesis. It has been proposed that metabolic changes during aging could be a driver for tumorigenesis [11]. This ‘Geroncogenesis’ hypothesis proposed that metabolic reprogramming occurs before tumorigenesis. It will be interesting to discuss the role of mitochondria, the hub of metabolism, in tumor origin.

Mitochondria convert dietary calories into ATP by oxidative phosphorylation (OXPHOS). The organelle plays an important role in supporting physiological activities, maintaining body temperature and participating in many metabolic functions such as fatty acid oxidation, biosynthesis of pyrimidines, haeme metabolism and Fe–S assembly [12]. Over the last decade, an overwhelming body of evidences indicated that mitochondria played a key role in regulating immune response [13]. Recently, mitochondria are also shown to be important for epigenetic regulation [14]. As the central hubs of cell metabolism, cell signaling and cell death, mitochondria are critically involved in aging [15]. *In vitro* and *in vivo* studies revealed that mitochondrial functions have been dramatically impaired during aging [16]. The contribution of mitochondrial stress to aging has also been widely discussed [17].

Mitochondrion is the only organelle possessing its own DNA in animals, and mtDNA encodes essential proteins of electron transport chain (ETC). MtDNA is essential in maintenance and regulation of mitochondrial functions, but is highly susceptible to replication cycle defects, damage induced by ROS and lack of mtDNA repair mechanisms. MtDNA could exist in hundreds to thousands of copies. After mutation occurs in one of many mtDNA copies in a cell, it is always functionally recessive at the beginning even it is a very pathogenic mutation. The mutated molecule undergoes random walking in subsequent cell division or mitochondrial turnover, a process that is similar to evolutionary dynamics of a new mutation in an asexual population. Concepts and approaches in population genetics can be applied to understand fate of mtDNA mutations during aging, and it has been shown that a portion of new somatic mutations could take decades to reach to frequency high enough to have functional consequences [18, 19].

Cancers occur in a progressive course, indicating that the most important risk factor contributing to such metabolic changes should be age-related. For chromosomal genes, only two copies are present in most of the cell types, so it is difficult for Mendelian genetics to provide the required aging clock for metabolic decline [2]. In contrast, the unique features in mitochondrial genetic system can be a candidate underlying age-related metabolic decline. As mtDNA mutations accumulate with age, mutation load constantly increases with direct metabolic consequences, followed by progressive loss of cellular functions, ultimately resulting in age-related phenotypes. Thus, accumulation of mtDNA mutations can be used as a proxy of ‘aging clock’ in decline of metabolic function. Here, we focus on mtDNA mutations and summarize literature indicating that accumulated mtDNA mutations during aging, and resulting metabolic reprogramming, nuclear genome instability and immune response could play important roles in tumor origin. The theme has enjoyed excellent coverage in the past [20, 21], but remains controversial, mostly as a result of technological limitations in tools related to mtDNA research. Here we will summarize recent development in related fields. The possible role of mtDNA mutations and their dynamics in determining cancer cell behaviors after tumorigenesis will not be discussed in this essay.

## MtDNA mutations increase with aging

**Figure 1.**
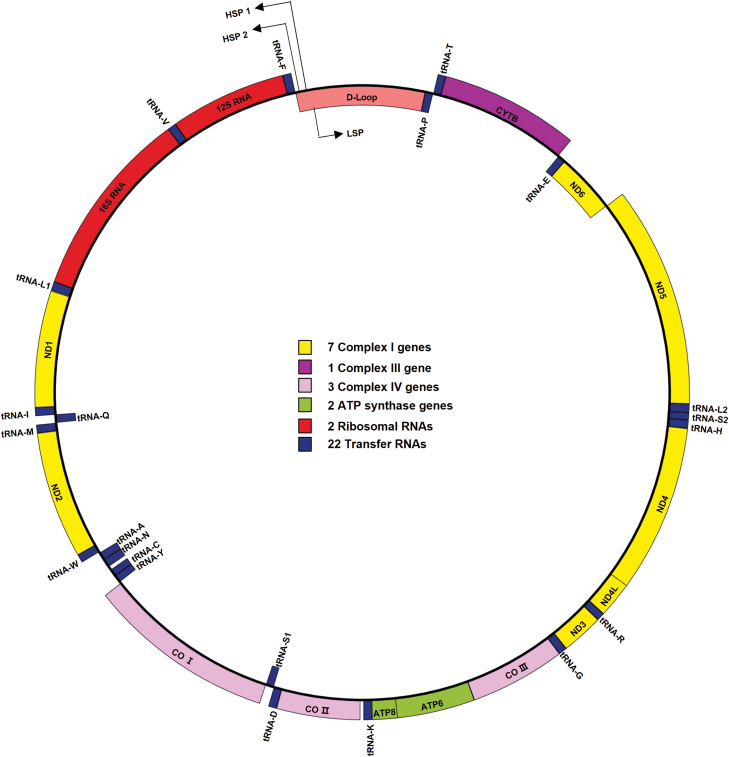
Human mitochondrial genome map. Schematic diagram of the 16,569 base pairs, circular, double-stranded mtDNA molecule. The components in the outer circle and the inner circle represent the genes encoded by the heavy strand and the light strand, respectively. As shown, the genes encoding the mitochondrial respiratory chain: *ND1-4, 4L, 5, 6*, *COI-III, ATP6* and *ATP8*, *Cyto B*; the two ribosomal RNAs (red boxes) and 22 tRNAs (blue boxes). Genes transcribed from HSP include 14 tRNAs, 2 rRNAs and 12 mRNAs, whereas LSP drives the transcription of the remaining 8 tRNAs and *ND6*. The figure was generated by Organellar Genome DRAW (OGDRAW) (https://chlorobox.mpimp-golm.mpg.de/OGDraw.html).

Human mtDNA is a double-stranded, circular genome with a size of 16,569 bp. During endosymbiosis in the past 2–4 billion years, most genes encoded by mitochondrial genome were lost or transferred to nuclear genome. At present, mtDNA only preserves genes that are required for mitochondrial protein synthesis, including 2 rRNA genes, 22 tRNA genes, and 13 polypeptides encoding genes (Fig. 1) [22]. Besides these 37 genes, there is a 1.2 kb non-coding region (NCR) in mtDNA, which contains elements like promoters of both strands and the origin of heavy-strand replication that are essential for transcription and replication of mitochondrial genome. Several recent studies found that mtDNA also encodes short peptides, which can translocate to nucleus to regulate nuclear gene expression under metabolic stress [23, 24]. The remaining ~1200 mitochondrial proteins are encoded by nuclear DNA.

**Figure 2.**
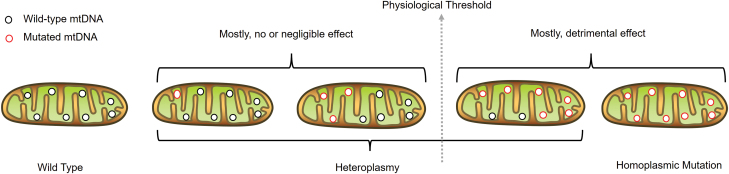
Different mtDNA mutation states. When no mutation is present, all mtDNAs in a cell are identical (wild-type homoplasmy). When mutations occur, the mutated mtDNA will coexist with the wild-type mtDNA, which is termed as mtDNA heteroplasmy. Most mtDNA mutations are functionally recessive. New mutations can clonally expand to a higher level up to or over the physiological threshold due to random walking process, then manifesting function defects. Furthermore, mtDNA mutation could be fixed in mtDNA population, leading to homoplasmic mutation.

A strong association between human aging and mtDNA mutations has been observed over the past few decades [25–27]. By far, mtDNA primarily suffers from two types of mutations: (a) single-site mutations, including transversion and transition; and (b) fragment rearrangements, such as mtDNA insertions and deletions. There are multiple mtDNA copies within a single cell. In most instances, mutations are only harbored by part of mtDNA copies, so that mutated mtDNA copies coexist with wild-type mtDNA, a phenomenon termed as heteroplasmy (Fig. 2). The ratio of mutated mtDNA among all mtDNA copies is called heteroplasmy frequency or heteroplasmy level. Due to functional redundancy, mtDNA mutations need to reach to a certain threshold for functional consequences. A strict functional threshold, however, could be misleading because changes in heteroplasmic levels can cause different functional consequences even for the same mutations [28]. High mutation rate of mtDNA (nearly 10–100 folds of nuclear DNA) can lead to accelerated mutation accumulation during aging [27, 29]. For example, there is a high level of mtDNA fragment deletion in aged substantia nigra neurons, associated with respiratory chain deficiency [30]. Point mutations in mtDNA are significantly accumulated in aged mitochondria compared to young mitochondria from mouse brain synaptosomes, resulting in reduced mitochondrial function, and severe respiratory deficiencies [31]. In addition, frequency of mtDNA point mutations increases nearly five-fold during a longer lifespan in human [32]. Since mtDNA mutations can accumulate with age, their effects are expected to grow because human lifespan has increased significantly over the past decades.

**Figure 3.**
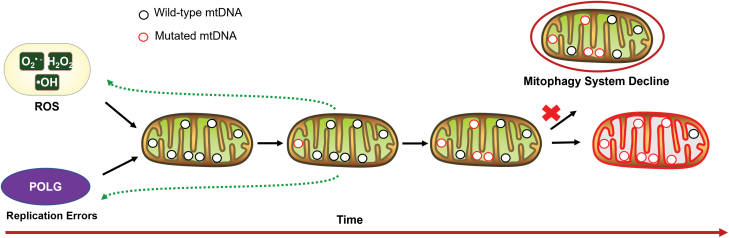
Mechanisms for accumulation of mtDNA mutations during aging. Cellular ROS and replication errors contribute to new mtDNA mutations. Mutated mtDNA could cause OXPHOS defects, enhancing ROS generation; mutated mtDNAs also provide inaccurate templates for replication, which sometime could be positively selected. Furthermore, less effective mitophagy during aging will aggravate accumulation of mtDNA mutations. Eventually, mtDNA heteroplasmy level will exceed a certain threshold, inducing pathogenic effects.

Multiple mechanisms can be responsible for mtDNA mutations. ROS has been considered as an important cause of mtDNA mutations for a long time [33]. The fact that mtDNA is replicated more frequently than nuclear genome may be another source inducing mtDNA mutations. Indeed, research with more genomic sequence data indicates that replication error could be more important in generating new mtDNA mutations [34]. Interestingly, the unique machinery of mtDNA replication could also facilitate expansion of damaged mtDNA [29]. Mitochondrial autophagy (mitophagy), the system responsible for eliminating damaged mitochondria, also undergoes functional decline during aging [35], which may accelerate the accumulation of mtDNA mutations (Fig. 3).

### Increased oxidative stress during aging induces mtDNA mutations

ROS are by-products of physiological cellular processes, primarily generated by electron leakage across electron transfer chain (ETC). Because physical location of mtDNA is close to ETC, ROS-induced lesions and instability in mtDNA have been conventionally considered as major sources of mtDNA mutations. Indeed, oxidative damages accumulated in mtDNA during normal aging have been reported to be a risk factor for age-related disorders [20]. ROS could cause DNA damage by means of single nucleotide alterations, single and double-strand breaks, sugar modification damages, all of which can generate errors in DNA templates [36]. 8-oxo-dG has been considered the most common mutation induced by ROS. In this circumstance, an A base will be introduced to pair with an oxidized G, resulting in a G: C to T: A transversion [37]. Owing to a lack of histone protection and a less effective DNA repair system, mtDNA is more vulnerable to this damage than nuclear DNA. However, recent studies suggest that ROS damage may not be the major contributor to mtDNA mutagenesis during aging, because most observed mutation types in mtDNA of somatic tissues are transition from G: C to A: T, rather than ROS-induced transversion from G: C to T: A [32, 34].

### MtDNA replication facilitates accumulation of mtDNA mutations

Recent evidences suggest that most age-related mtDNA mutations are caused by errors during mtDNA replication, rather than unrepaired damage by ROS [32, 34]. Unlike nuclear DNA, mtDNA replication is not governed by cell cycle, and continuously occurs even in non-dividing cells [38]. In mitochondria, mtDNA is replicated in a replisome constituted of polymerase gamma, which is the only polymerase responsible for mtDNA replication. Polymerase gamma is a heterodimeric enzyme composed of a catalytic core and two accessory subunits. The catalytic subunit encoded by *Poly A* harbors regions with different functions: 1) an exonuclease domain with DNA polymerase activity; 2) a linker region with 3ʹ–5ʹ proofreading exonuclease activity; and 3) a polymerase domain with apyrimidinic activity required for enzymatic DNA repair [39]. The accessory subunits encoded by *Poly B* are necessary for mtDNA replication by promoting progressive DNA synthesis [40]. Polymerase gamma possesses high fidelity, however, given the high volume of mtDNA replication required over lifetime in cells, base substitution errors are introduced more often than in nuclear genome [41]. A study on aging also suggested that predominant transition mutations may be induced by misincorporation of DNA polymerase gamma [34]. Indeed, even in post-mitotic cells such as neurons, which contain about 10,000 mtDNA molecules within a single cell, mtDNAs need to be replicated almost 3000 times for a person with a lifespan of 80 years. The higher frequency of replication exacerbates mutation vulnerability of mtDNA [42].

Two other mechanisms that are related to replication could also contribute to mtDNA mutation accumulation during aging. 1) compared to nuclear DNA polymerase, mtDNA polymerase grammar is more likely to be damaged by ROS owing to its specific location. Mutations in mtDNA polymerase gamma may affect its fidelity, and increase vulnerability of mtDNA [12]. Inherited polymerase mutations were also observed, albeit at a low frequency [29]. Studies *in vitro* also demonstrated that expressing a proofreading-deficient version of polymerase grammar dramatically increased somatic mtDNA mutations [43]. 2) due to limitations of mtDNA repair system, double-stranded break repairing would form fragment rearrangement in mtDNA, causing insertions and deletions (indel) in the template [44]. Although mtDNA-encoded genes are essential for ETC, replication of mtDNA will not be affected by most mtDNA mutations. Therefore, mtDNA with large fragment indels could still be replicated and transmitted to daughter mitochondria. In some instances, mutated mtDNA, especially with deletion, could even be replicated preferentially [45]. In addition to the above mechanisms, many studies have documented that mutation incidence in mtDNA d-loop was higher than that in coding regions [31, 46]. Whether this reflects bias in mutational process, or survival of mutations, or both, remains to be further investigated. Nevertheless, since the d-loop region contains the origin of heavy-strand synthesis (O_H_) and the site of mtDNA transcription promoters (HSP1, HSP2, and LSP), mutations in this region will affect mtDNA replication and transcription, potentially leading to more mutations.

### Dysfunction of mitophagy increases level of mtDNA mutations

A growing body of evidences supports that general autophagy has an anti-aging role [47, 48]. Mitophagy, as a specific autophagy process, mediates removal of dysfunctional mitochondria [49]. The PINK1/Parkin pathway is one of the most studied mitophagy pathways. PINK1, a mitochondrial kinase, is selectively stabilized on mitochondrial surface. When mitochondria are damaged and lose membrane potential, PINK1 will recruit E3 ubiquitin ligase Parkin to ubiquitinate mitochondrial outer membrane proteins, inducing autophagic elimination of damaged mitochondria [50]. Beyond the role in Parkinson’s disease, Parkin has also been found to be a tumor suppressor, suggesting a potential function of mitophagy in cancer biology [51]. Besides the PINK1/Parkin pathway, many other mitophagy pathways have also been recently discovered [52]. Recently, a migrasome-mediated mitochondrial quality-control process, mitocytosis, has been reported [53], but is largely limited in migrating cells and the effect is slight compared to mitophagy. Mitophagy has been considered as the most powerful tool to eliminate mtDNA mutations, especially in aged tissues where mtDNA mutations may accumulate at an extensive level.

A number of studies have documented that mitochondrial dynamics, including fission and fusion, are closely related to mitophagy. Mitochondrial fission and fusion processes are mediated by large guanosine triphosphatases (GTPases). For instance, mitofusin (*Mfn*) proteins mediate the fusion of mitochondrial outer membrane, and mitochondrial fission is regulated by Dynamin-related protein 1 (*Drp1*) [54]. Several studies indicate that Parkin-mediated turnover of Mfn can disrupt the balance of mitochondrial dynamics, leading to decreased fusion and increased fission [55, 56]. These findings suggest that mitochondrial fission can promote the segregation of damaged mitochondria and facilitate their mitophagic clearance. However, mitochondrial fusion may have the reverse effect on the clearance of mtDNA damage. Through fusion, mitochondria with mutant mtDNA can remain functional by exchange of non-mutant mitochondria or different mutant mitochondria, where the missing component is in excess, compensate for their deficiencies via sharing mtDNA, proteins and lipids, thereby rescuing the mitochondria with mutant mtDNA and mitigating the effects of moderate environmental stresses [57–60].

When mitophagy is triggered, lysosome will wrap up and degrade damaged mitochondria [61]. The mitochondrial-lysosomal axis theory of aging suggests that overload of damaged mitochondria in lysosomes will lead to the crash of cellular cleaning system in aged cells [62]. Consistent with this theory, an increase of lysosomal contents was observed in replicative aged human endothelial cells [63]. Lysosome also contacts the mark sites of mitochondrial fission and regulates mitochondrial dynamics [64]. Within the aging context, lysosome dysfunction may influence fission, then further affect mitophagy. It is important to note that lysosome-mediated degradation is not always effective, for example, some mitochondria with mtDNA mutations and deletions exhibit reduced respiratory activities but normal membrane potential, resulting in less lysosomal degradation [65].

Recent studies have reported that mitophagy declines during aging in mammals [35]. Functionally compromised mitophagy can accumulate dysfunctional mitochondria [66], which could exacerbate mtDNA mutation accumulation during aging. These observations indicate a potential route for health intervention. When mitophagy was promoted by overexpression of Parkin in *Drosophila*, aging phenotypes were postponed and lifespan increased significantly [67]. Furthermore, overexpression of Parkin in cybrids can eliminate mitochondria with deleterious mutations in *COI*, enrich mitochondria with wild-type mtDNA, and restore function of ETC [65]. Dietary urolithin A could also prevent age-related accumulation of dysfunctional mitochondria and prolong the lifespan in *C. elegans* by facilitating mitophagy [68]. Interestingly, enhanced *Drp1* expression, by increasing mitochondrial fission and promoting mitophagy, can also prolong lifespan in *Drosophila melanogaster* [69, 70].

## High-frequency mtDNA mutations in single-cells

**Figure 4.**
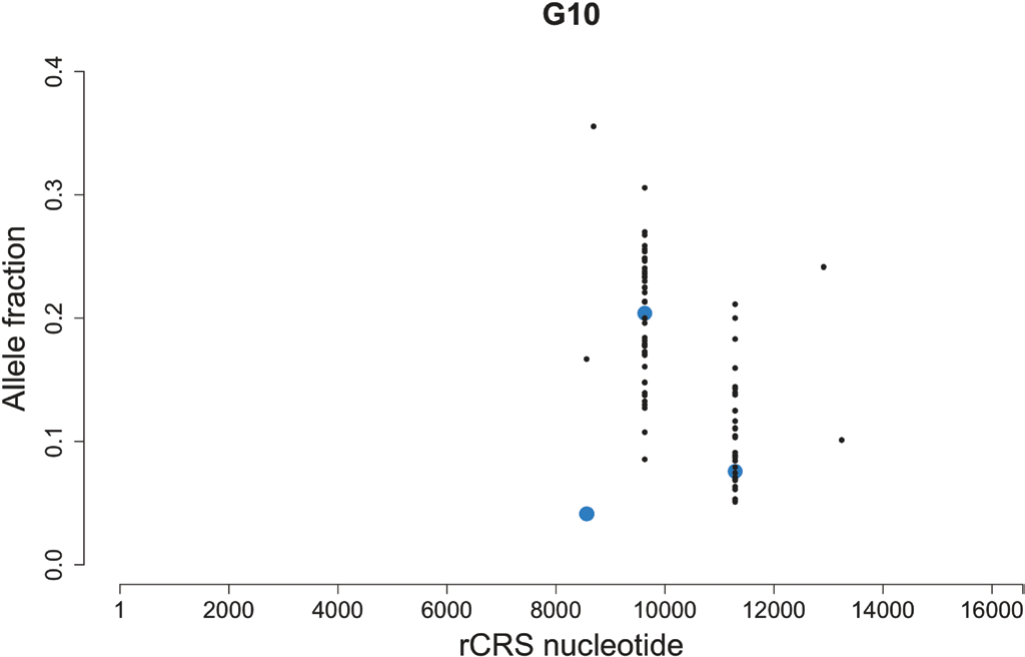
Heteroplasmic variants and their allele fractions identified from bulk ATAC-seq data (blue) and single-cell ATAC-seq (black) of a TF-1 cell population (ID: G10). Raw fastq was obtained from Ludwig et al. [74].

Investigation on the impact of mtDNA mutations on aging and age-related disease is limited by difficulties in accurately estimating prevalence of mtDNA heteroplasmy. Technological innovations allow mtDNA variant identification at low frequencies. Analysis of high-depth resequencing data of human mtDNA revealed heteroplasmic variants scattered throughout mtDNA genome [71]. It is interesting that mtDNA heteroplasmy has been observed in almost every human subject, with a frequency between 0.5% and 1.5% [72]. However, these observations are very limited and only represent a very small amount of mtDNA mutations. Typically, all mutations are generated on a single mtDNA molecule, but mutations are detectable only when mutated mtDNA reaches a particular level [73]. Recently, several groups have reported that scATAC-seq (single-cell Assay for Transposase-Accessible Chromatin with high-throughput sequencing) data can be used to identify single-cell mtDNA mutations [74–76]. An illustrative comparison of mtDNA mutation identified from single-cell versus bulk ATAC-seq of the same TF-1 cell line is shown in Fig. 4. Apparently, all mtDNA mutations observed at the population level were also detectable in many single-cells. Their allele fractions in single-cells, however, varied dramatically, with the population level being the average of all single-cells. On the other hand, some mtDNA mutations were singletons, being uniquely carried by only one cell in the population and were not detectable with bulk-sample ATAC-seq.

**Figure 5.**
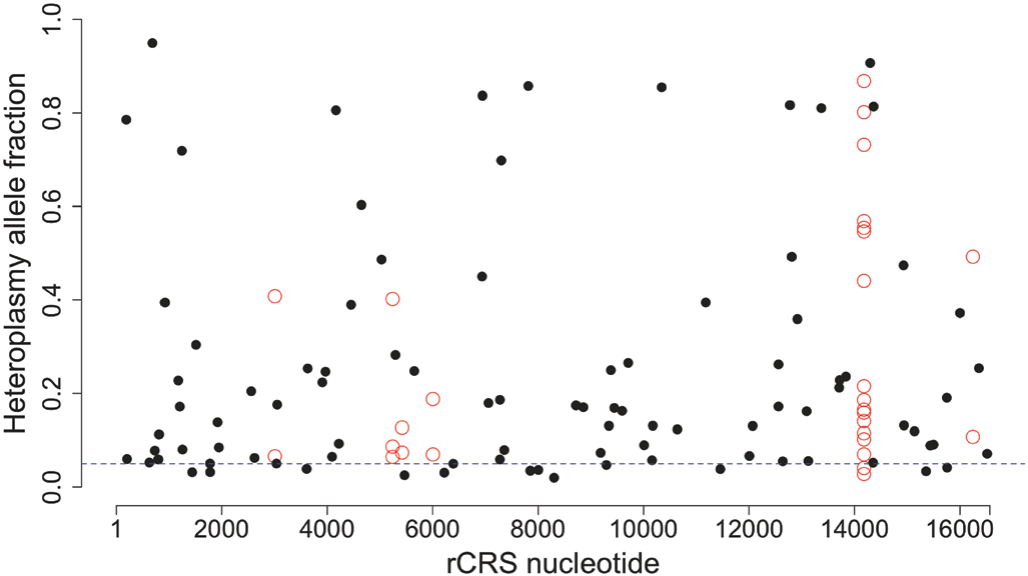
Heteroplasimc variants and their allele fractions were identified by single-cell ATAC-seq in common myeloid progenitors from a 53-year-old female (ID: BM1077). Black solid dots represent variants that are detected in only one cell, and red hollow circles represent variants that are detected in multiple cells. Raw fastq was obtained from Buenrostro et al. [77].

It is more difficult to detect somatic mtDNA mutations. Common mtDNA mutations carried by most cells likely represent germline or somatic mutations that exist in early life, whereas rare or singleton mutations likely arise somatically later in life and in a tissue-specific manner [74]. Due to genetic drift and/or natural selection, mtDNA heteroplasmy frequency in cells can change over time [73]. Burgstaller et al. [77] reported that the variance of a heteroplasmic variant frequency increases linearly with age in oocytes of female mice and somatic cells of pups. Single-cell mtDNA sequencing can help reveal common mutations and identify rare mutations masked by the bulk population, not only across cells but also over time, at high resolution. Indeed, many high-frequency mtDNA mutations can be observed *in vivo* only at the single-cell level, even for mid-aged individuals (Fig. 5). The ongoing development in single-cell technology will allow us to detect mtDNA mutation dynamics more precisely throughout human lifespan in the near future.

## MtDNA mutations can contribute to tumorigenesis

Increasing evidences suggested that mitochondria may play a central role in the etiology and pathogenesis of tumorigenesis [20, 33]. MtDNA encodes proteins that are important for mitochondrial functions but is highly susceptible to mutations. It is generally believed that low-frequency mtDNA heteroplasmic mutations remain functionally recessive. The observed low-frequency mtDNA mutations in bulk mtDNA sequencing could lead to the misconception that they are functionally not important. However, abundant mtDNA mutation input and subsequent random drift during aging could lead to different high-frequency mutations in different cells. Because most regions in mtDNA encode core functions related to mitochondria, different mtDNA mutations in individual cells can likely induce pathological effects in mitochondria-related functions, such as metabolism, epigenetic regulation, immune response, and cell death. Due to high energy demand, neurons are highly dependent on mitochondria, and mutations in mtDNA can be an important reason underlying mitochondrial functional decline in neurons, and thus neurodegenerative diseases [78, 79]. However, in this review, we will focus on mtDNA mutations and their possible role in tumor origin.

In recent years, a substantial number of mtDNA mutations, including point mutations and deletions, have been identified in human cancer mutation spectrum [26, 80]. It remains to be investigated whether such observation is causal or correlative. Metabolic changes, nuclear genome instability and inflammation have all been discussed as direct causes leading to tumorigenesis [81–83]. Considering the specific contribution of aging to cancer origin and mtDNA mutation dynamics during aging, it could be fruitful to contemplate the role of mtDNA mutation accumulation with aging in tumorigenesis. Here, we will discuss how mtDNA mutations can induce cellular metabolic changes, nuclear genome instability, and immune response.

### MtDNA mutations lead to metabolic reprogramming

**Figure 6.**
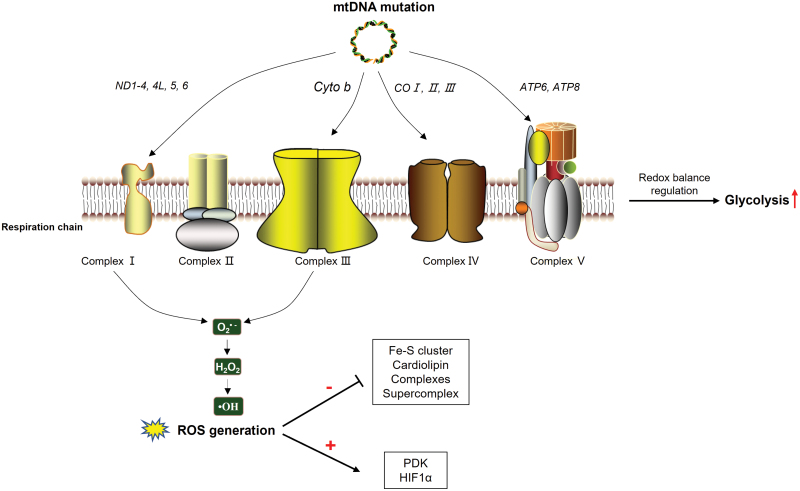
MtDNA mutations cause OXPHOS defects and induce oxidative stress, contributing to tumorigenesis. Proteins encoded by mtDNA participate in respiratory chain complex assembly, and mtDNA mutations will impair OXPHOS, reprogram cellular metabolism toward to glycolysis, and increase ROS generation. ROS could damage several critical enzymes involved in metabolism, further damaging mitochondrial function. Moreover, ROS could activate hypoxia-inducible factor activity 1α (HIF1α), favoring metabolic reprogramming.

Metabolic reprogramming is one of the most important hallmarks of cancer. It was even hypothesized that persistent metabolic reprogramming could proceed to tumorigenesis [84]. Cancer cells have largely been characterized by declined oxidative phosphorylation and enhanced aerobic glycolysis, a phenomenon observed by Warburg almost a century ago [85]. Proteins encoded by mtDNA are core components of ETC complexes, whereas the d-loop region, tRNAs and rRNAs are all directly involved in mtDNA replication, transcription, or translation. Accumulated mtDNA mutations could compromise mitochondrial oxidative metabolism, and the defective ETC causes inefficient electron transfer, promoting ROS generation. ROS may damage mitochondria and mtDNA, and decrease ETC efficiency, resulting in a vicious circle of mitochondrial functional decline. We will focus our discussion on OXPHOS defects and ROS production induced by mtDNA mutations (Fig. 6), it is nonetheless worth noting that almost all aspects of metabolisms can be affected by mtDNA mutations.

#### MtDNA mutations induce OXPHOS defects and glycolytic reprogramming in tumorigenesis

Mutations in mtDNA could cause impaired OXPHOS activity, which may trigger glycolysis for cellular energy compensation. Complex I is the largest component in respiratory chain, in which seven subunits (*ND1-4*, *4L*, *5*, *6*) are encoded by mtDNA (Fig. 1). Defects in complex I are the most common cause of oxidative phosphorylation disorders, which are associated with diverse clinical symptoms [86]. Many studies have documented that complex I deficiency induced by mtDNA mutations is involved in cellular metabolic reprogramming towards glycolysis [81]. Using the transmitochondrial cybrid cell method, tumorigenic effect of mutations in mtDNA-encoded complex I subunits has been partially elucidated. Under the same nuclear DNA background, cybrids carrying mtDNA mutations in *ND2* and *ND5* subunits showed lower oxidative phosphorylation, higher levels of lactate production, as well as higher tumorigenesis potentials [87, 88]. In complex III, *cyto b* was the only subunit encoded by mtDNA. *Cyto b* mutations have been indicated in different cancers by enhancing lactate production and tumor growth [89]. Complex IV is the last electron acceptor. Three catalytic subunits (*COI*, *II*, *III*) of complex IV are encoded by mtDNA (Fig. 1). Cybrids carrying mutations in *COI* showed decreased respiratory activity, higher proliferation rate and increased tumor growth *in vivo* [90]. Complex V, also called mitochondrial ATP synthase, is the last enzyme of respiratory chain. The enzyme utilizes proton gradient across mitochondrial inner membrane to drive ATP synthesis. *ATP6* and *ATP8,* two subunits of complex V are encoded by mtDNA. Cybrids harboring a functional mutation in *ATP6* can confer growth advantage in tumors [91].

MtDNA mutations accumulated during age could occur extensively across whole mitochondrial genome, inducing compromised OXPHOS functions. A recent study found that tumor volume was larger in OXPHOS-deficient mice than that in normal group, and the difference was caused by age-related mtDNA mutations, accelerated cell proliferation, and reduced apoptosis. Of note, these mutations are distributed across the entire mtDNA genome [92]. In response to OXPHOS deficiency, cells also upregulated serine synthesis pathway, which is critical to support cell proliferation and survival in cancer development, providing a metabolic advantage for tumorigenesis [92]. Indeed, mitochondrial energetic defects induced by mtDNA mutations may force a compensatory growth advantage, thus protecting cells from apoptosis and contributing to tumorigenesis [87].

#### MtDNA mutations induce redox imbalance in tumorigenesis

Mitochondrial OXPHOS process consumes more than 90% of intracellular oxygen. Electrons from NADH and FADH2 are delivered to molecular oxygen through complex I to IV, generating water and proton gradient. In general, complex I and complex III are primary sources of ROS. NADPH oxidase (NOXs) is also a leading site for ROS generation. Of note, compromised respiratory chain could activate NOXs, further enhancing cellular ROS levels [93]. We will focus our discussion on mtDNA mutations and ROS production in this section. More than 30 years ago, Denham Harman first proposed that aging resulted from accumulation of mitochondrial oxidative damages induced by ROS [94]. The theory remains controversial since ROS is involved in a lot of important signal transduction pathways [95]. Meanwhile, it is important to note that excessive ROS production and mitochondrial functional defects are not equivalent to each other, as the former is only one of many consequences from the latter.

Under physiological conditions, most oxygen is effectively reduced to water, and only 1%–2% oxygen is reduced to superoxide radicals (O_2_^-^) at complex I and III [96]. Superoxide radicals in mitochondrial matrix could be converted into peroxide (H_2_O_2_) by mitochondrial superoxide dismutase 2 (*SOD2*), then released into mitochondrial intermembrane space and cytosol. H_2_O_2_ could be further turned into hydroxyl radical (OH) [97]. Superoxide, peroxide and hydroxyl radical are the three primary sources of cellular ROS (Fig. 6). Under ETC depression induced by disruptive mtDNA mutations, electron leakage is enhanced and generation of ROS is elevated, which occurs in many cancer cells [98]. As a toxic by-product of OXPHOS, excessive ROS could act as mutagens and cellular mitogens, and is highly involved in tumorigenesis and cancer development. Excessive ROS may further lead to mitochondrial dysfunction through multiple mechanisms, and the following two general aspects are of particular importance.

(1) ROS could directly disturb activities of several key enzymes involved in mitochondrial metabolism, such as Fe-S cluster, NADH dehydrogenase, and SDH, resulting in inactivation and inhibition of intermediates generation [99]. This may further stimulate mitochondrial dysfunction and metabolic rewiring. Enhanced ROS generation would cause supercomplex disorganization, which might lead to further deleterious consequences, such as alteration of electron transfer and proton translocation [100]. The abundance of supercomplex depends on mitochondrial phospholipid content, and supercomplex assembly may limit the extent of ROS generation through respiratory chain [101]. Interestingly, ROS could oxidize mitochondrial phospholipids, especially cardiolipin. Cardiolipin, as a unique mitochondrial inner membrane lipid, could stabilize ETC supercomplex as well as individual complexes. Mitochondrial ETC activity could be significantly influenced by exposure to ROS via oxidative damage of cardiolipin [102].
(2) ROS can induce pyruvate accumulation by stimulating pyruvate dehydrogenase kinase (PDK) [103], resulting in reduced pyruvate flux into TCA cycle, which can influence metabolic intermediate levels, and consequently activate HIF1α [81]. Under physiological condition, HIF1α is maintained at an inactivation state, which may inhibit expression of genes involved in glycolysis and pro-tumorigenesis. Activation of HIF1α enhances PDK expression and PDK inactivates TCA cycle enzyme including pyruvate dehydrogenase (PDH), which decreases levels of NADH and pyruvate and forces metabolic reprogramming towards aerobic glycolysis [104]. Thus, mtDNA mutations could lead to a pseudohypoxia response by activation of HIF1α, which could remodel cellular metabolism and facilitate tumorigenesis.

### MtDNA mutations induce genome instability

**Figure 7.**
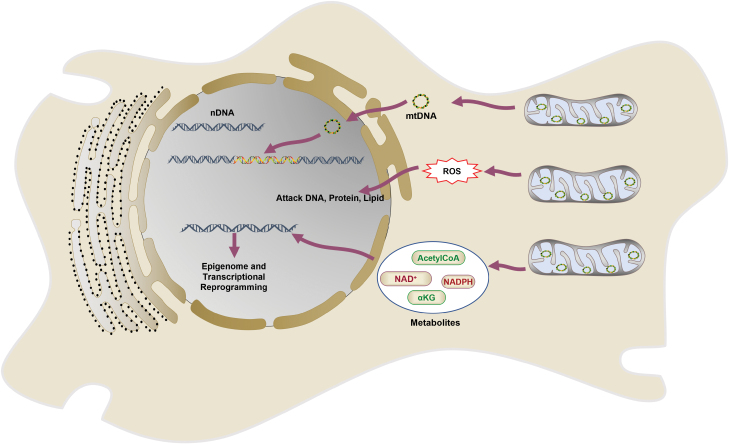
Mechanisms for mtDNA mutation-induced genome instability. Firstly, mtDNA mutations contribute to ROS generation. ROS could directly damage DNAs, proteins, and lipids, thus leading to genomic instability. Secondly, mtDNA could be transferred into nucleus and embedded within nuclear genome, resulting in genomic instability. Thirdly, mtDNA mutations could alter metabolism and influence cellular concentrations of metabolic intermediates, which can induce epigenomic and transcriptional reprogramming, and eventually result in genome instability.

Nuclear genome instability, a hallmark of aging and cancer, is considered to be an early event in tumorigenesis. Generally, three types of genome alterations have been implicated: (a) chemical damage to nuclear genome, including both single-strand and double-strand breaks of DNA; (b) DNA mutations, such as insertion, deletion, or single nucleotide substitution; and (c) epigenetic perturbations which can affect gene regulation without changes in DNA sequence [105]. MtDNA mutations showed a profound impact on the instability of nuclear genome for all these three levels (Fig. 7). By increasing ROS generation, mtDNA mutations could induce chemical damage to nuclear genome. Moreover, mtDNA fragments could be inserted into and restructure nuclear genome. More and more evidences indicate that mtDNA mutations could change epigenetic regulation of nuclear genome.

It will be fruitful to illustrate more mechanisms directly linking mtDNA mutations and nuclear genome instability. It is worth pointing out that by increasing genomic instability, somatic cells could increase their odds in acquiring phenotypes that increase their ability to proliferate, migrate, colonize ectopic sites, survive hostile tissue environments, and evade attack by immune system. These phenotypes are, of course, hallmarks of lethal cancers. Interestingly, similar arguments have been made in an evolutionary context, where a mutator phenotype can enable its host to better survive environmental stress [106, 107].

#### MtDNA mutations promote ROS generation

Oxidative DNA damage has been considered as an important inducer of cancer [108, 109]. As we discussed in previous sections, accumulated mtDNA mutations promote ROS generation, which was considered as a major source of nuclear DNA damage [110]. ROS acts as mutagen and causes various types of DNA damage such as single nucleotide substitutions, double- and single-strand breaks [111]. ROS slows down replication fork by dissociation of PRDX2 and TIMELESS [112]. ROS oxidized nucleotides interfere with replication fork by affecting polymerase activity [113]. Replication of damaged DNA without repair leads to increased DNA mutations [114]. In response to accumulated DNA damage, DNA damage response (DDR) will be triggered to maintain genome integrity by promoting apoptosis or cell cycle arrest [115]. DDR will also promote DNA and chromatin repair. New mutations may be introduced during repair, which can potentially favor uncontrolled cell proliferation, and subsequently induce tumorigenesis [116]. In response to increased ROS level, the antioxidant system will be enhanced, which may also induce genomic instability, and allow cancer cells to survive under oxidative stress, both of which may exacerbate cancer development [33].

#### MtDNA translocations reconstruct nuclear genome

Natural transfer of mtDNA into nuclear genomes is an evolutionarily ongoing process. The mtDNA sequences in nuclear genome are described as NUMTs (nuclear mtDNA segments) [117]. Different technologies have been applied to investigate how mtDNA translocates into nuclear genome. Unexpectedly, large mtDNA fragments, in some cases, even entire mtDNA sequence, have been observed to integrate into nuclear genome of tumor cells [118]. Compared to blood samples, tumor samples from the same individuals showed a four-fold elevation of NUMTs in nuclear genome [119]. A recent study found that somatically translocated mtDNA were extensively rearranged, associated with increased numbers of structural rearrangement events within nuclear genome, implying that nuclear genome may experience high instability during mtDNA translocation events [19]. Therefore, increased mtDNA translocation events have been considered to contribute to tumorigenesis due to their impact on genomic instability. The detailed mechanism underlying NUMTs formation and their exact role in human cancer development remain to be elucidated.

#### MtDNA variants influence epigenetic regulation of nuclear genome

More and more evidences indicate that mitochondria play an important role in influencing nuclear genome epigenetic regulation [14, 120]. Indeed, as the center for cellular metabolisms, mitochondria provide necessary metabolites in modifying different types of biomolecules, including DNA, RNA, and histone markers. Changes in mtDNA sequence could conceptually lead to downstream changes in these epigenetic regulations. Indeed, it has been reported that mtDNA genetic background is associated with susceptibility to certain cancer types [121]. An epidemiological study has documented that haplogroups U showed about 2 to 2.5-fold higher risk for renal and prostate cancer [122]. These association analyses suggest that specific mtDNA variants could regulate nuclear genome expression to drive oncogenic transformation. In a study using *trans*-mitochondrial mice, the authors showed that different mtDNA haplotypes could deferentially regulate α-ketoglutarate to catalyze transition from 5-methylcytosine to 5-hydroxymethylcytosine, induce distinct DNA methylation signatures and further influence gene expression patterns [123]. A progressive increase of human mtDNA heteroplasmy could cause distinctive epigenomic changes by affecting nuclear histone acetylation, which further induces transcriptional reprogramming of nuclear genome [28, 124]. Another study discovered a complicated relationship between mtDNA copy number and global nuclear DNA methylation [125]. Therefore, mtDNA variants or mutations could modify nuclear genome without changing DNA sequence, which can induce genome instability. More studies are needed to further investigate epigenetic mechanisms underlying the impact of mtDNA mutations on nuclear genome instability.

#### Other mechanisms from mitochondrial dysfunction to genome instability

Besides directly altering nuclear genome, mtDNA mutations could lead to genome instability by other mechanisms. For example, mtDNA mutations could impair proteins involved in nuclear genome maintenance. It has been shown that impairment of mitochondrial functions could break assembly of iron-sulfur clusters (ISC) and induce loss of heterozygosity (LOH), which is an important form of genomic instability in tumor regression [126]. High-frequency of mtDNA replication can also sequester nucleotides to mitochondria, and decrease dNTP availability for nuclear genome replication, thus compromising maintenance of nuclear genome [127]. Cellular availability of NAD+, an important coenzyme for genome stability modulator, Ppar1, is also regulated by mitochondrial functions [128, 129].

### Mitochondria prime immune response

**Figure 8.**
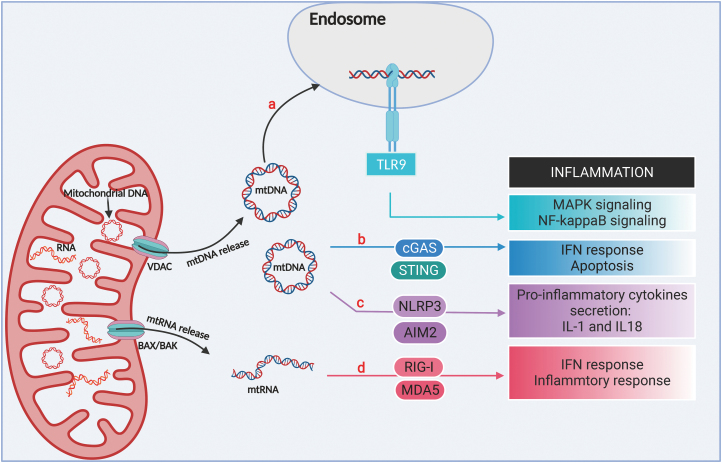
Overview of immune response triggered by mtDNA and mtRNA. Leakage of mtDNA can be detected by various DNA sensors including TLR9, cGAS-STING, and NLRP3 inflammasome, which trigger immune responses. (a) mtDNA binds to TLR9 in endosome to activate NF-kB signaling pathway; (b) cGAS recognizes mtDNA in the cytoplasm and activates STING to trigger Type-I interferon signaling and the downstream IRF3-dependent apoptosis; (c) NLRP3 inflammasome can recognize oxidized mtDNA to activate caspase-1 and the maturation of pro-inflammatory cytokines secretion; (d) mtRNA recognized by RNA sensor RIG-I and MDA5 to elite Type-I interferon signaling and pro-inflammatory cytokines secretion. Created with BioRender.com.

Chronically activated immune response and inflammation have been argued as causes of cancer origin [83, 130]. Inflammation increases with age even without pathogen participation. Mitochondria are cellular organelles which orchestrate ranging biological processes including energy, metabolism, cell death and, interestingly, immune response [131]. Mitochondria evolved from a bacterial ancestor, and interestingly, mtDNAs still maintain certain structural properties even after 2 billion years of evolution. As a result, leakage of mtDNA can function as a damage-associated molecular pattern (DAMP), which is recognized by different pattern recognition receptors (PRRs) including cGAS-STING, TLR9 and NLRP3 inflammasome, and trigger the activation of immune responses [132–135]. Mitochondrial RNA (mtRNA) can also trigger cellular inflammation [136, 137]. All of these could be important reasons underlying non-pathogen related inflammation during aging (Fig. 8). Recent data have demonstrated that accumulation of damaged mitochondria evokes inflammation. In this section, we will discuss the molecular mechanisms of mtDNA and mtRNA recognition, and the role of mitochondrial leakage in immune response and inflammation development. We only focus on mtDNA and mtRNA in this essay, it is nonetheless worth noting that metabolites, such as ROS, upon mitochondrial leakage, also participate in cellular immune response [138].

#### MtDNA and TLR9 signaling pathway

Toll-Like Receptor 9 (TLR9) recognizes unmethylated CpG dinucleotides prevalent in bacterial and viral DNA to evoke inflammatory responses [139]. MtDNA contains unmethylated and hypomethylated CpG motif that is similar to bacterial DNA, which can be recognized by TLR9 in endosomal compartment [140]. The activation of TLR9 signaling pathway can result in MAPK and NF-κB signaling activity to trigger pro-inflammatory cytokines production and inflammatory response [141]. TLR9 is broadly expressed in immune cells and regulates physiological homeostasis in response to CpG nucleotides. Raising evidence shows TLR9 expression in non-immune cell types also play important role in pathological response [142, 143]. Liver is a metabolic organ and serves as the first line of defense against infection. Study has shown that TLR9 in liver is required for non-alcoholic steatohepatitis (NASH) development in mice, which was activated by mtDNA released from hepatocytes after liver injury [144]. Also, mammalian red blood cells express intracellular TLR9 to scavenge cell-free mitochondrial DNA (cf-mtDNA) to promote inflammatory response and anemia [145].

The function of TLR9 in cancer development has been particularly well studied. In liver cancer, hypoxia induces translocation of HMGB1 to cytosol and binds to mtDNA to activate TLR9 signaling pathway, which subsequently promotes tumor growth [146]. In tumor microenvironment, mtDNA released from necrotic tumor cells can be sensed by TLR9, which accelerates tumor recurrence after irradiation [147]. However, study has also shown that uptake of tumor-released cell-free mtDNA by TLR9 can evoke anti-tumor immune activity in dendritic cells (DCs) and tumor-specific CTLs [148]. Although it is in the early clinical development, the agonists of TLR9 have demonstrated potential for cancer treatment [149].

#### MtDNA and cGAS-STING signaling pathway

Cyclic GMP-AMP synthase (cGAS), an important DNA sensor, directly binds dsDNA to form a dimer and convert ATP and GTP into 2ʹ3ʹ-cyclic GMP-AMP (cGAMP), which activates stimulator of interferon genes (STING) [133]. TANK-binding kinase 1 (TBK1) is recruited and activated to elicit the anti-viral immune response by phosphorylation of the transcription factor interferon regulatory factor 3 (IRF3) [132]. cGAS primarily detects invading pathogenic DNA, therefore, leaked dsDNA from mitochondrial during cell damage or infection can be sensed by cGAS to evoke necessary type-I interferons and inflammatory cytokines production.

Accumulating evidence has revealed that chronic dysregulation of inflammation can lead to cancer progression [150, 151]. In aged cells, mtDNA mutations accumulation might lead to ROS production and mtDNA leakage. mtDNA might act as a critical message to stimulate cGAS-STING pathway and induce chronic inflammation, which result in inflammation-driven carcinogenesis. However, it has also been shown that activation of the cGAS-STING signaling plays a role in cellular senescence, which might be an important mechanism for suppressing tumorigenesis [152, 153]. cGAS- or STING-deficient MEFs show accelerated cell proliferation and attenuated senescent phenotype [154]. Activation of the cGAS-STING signaling in senescent cells by recognizing the aberrant cytosolic chromatin fragments leads to the production of senescence-associated secretory phenotype (SASP) factors to further promote cell senescence [155]. Taken together, activation of cGAS-STING signaling can upregulate IFN production to exert anti-tumor roles by innate immune response.

#### MtDNA and inflammasome signaling pathway

Inflammasomes are large complexes that trigger very potent inflammatory effects, including secretion of pro-inflammatory cytokines like interleukin-1β (IL-1β) and initiation of pyroptosis (a programmed lytic cell death) [156]. In most cases, inflammasomes rely on Nod-like receptors (NLRs) for complex assembly. NLRP1, NLRP3, and NLRC4 have been demonstrated to form inflammasomes that promote immune responses [157–159]. The NLRP3 inflammasome is of great interest, as mutations in NLRP3 gene are related to various diseases in which the inflammasome is under constitutive activation [160]. In addition, it is widely expressed in many immune cells and can be activated by a wide range of stimuli, ranging from pathogen components like bacterial toxins to non-microbial substances [158]. However, no evidence supports the direct binding of NLRP3 to these substances. The underlying mechanism of NLRP3 inflammasome activation requires further investigation. Once activated, NLRP3 assembles with ASC and procaspase-1, leading to autoactivation of caspase-1 and subsequent signal cascade [161]. The NLRP3 inflammasome lies at the center of inflammation in pathological progress and degenerative diseases.

The linkage between mitochondria and NLRP3 inflammasome was firstly demonstrated in the macrophage that mitochondrial reactive oxygen species (mtROS) can elicit NLRP3 activation [134]. It was suggested that mtROS induced the interaction between NEK7 and NLRP3, leading to NLRP3 inflammasome activation [162]. Notably, accumulating evidences highlight the crucial roles of mtDNA in NLRP3 inflammasome activation [163]. Activation of NLRP3 inflammasome requires the priming of TLR4 ligand to promote new mtDNA synthesis, leading to generation of oxidized mtDNA (ox-mtDNA) fragments [164]. Ox-mtDNA was proposed to serve as the NLRP3 ligand for the enhanced inflammatory responses. Interestingly, a recent study suggested that circulating cell-free mtDNA (ccf-mtDNA) from type 2 diabetes-induced AIM2 inflammasome activation in macrophages [165]. Therefore, mtDNA activated NLRP3 inflammasome can evoke a pathological link to age-related diseases, including cancer.

#### MtRNA and immune signaling pathway

Homeostasis and metabolism of mitochondria nucleic acids are important for preventing aberrant immune activation. In addition to mtDNA, mtRNA is newly identified as a key intracellular damage signal sensed by cytosolic sensors [136]. mtRNA generated by mtDNA with potential to form mtdsRNA with a free 5ʹp which was similar to bacteria mRNA. mtRNA released to the cytoplasm is recognized by RNA sensor RNA helicase retinoic acid inducible protein I (RIG-I) and melanoma differentiation associated gene 5 (MDA5), which activate MAVS and subsequent phosphorylation of TBK1 and IKB kinase-ε (IKKε) [166]. Phosphorylation of TBK1 and IKKε can therefore recruit and activate the transcriptional factors interferon regulatory factors 3 and 7 (IRF3/7) and NF-κB for further IFNs and pro-inflammatory cytokines expression [167].

Owing to the bacterial origin, bidirectional transcription of mtDNA can generate overlapping transcripts which form double-stranded RNA structures with potentially high immunogenicity [168]. Depletion of degradosome components mitochondrial RNA helicase SUV3 and polynucleotide phosphorylase PNPase result in mitochondrial dsRNA (mtdsRNA) accumulation and release into cytoplasm to elite type-I interferon response in cells [169]. Moreover, silencing of BAX/BAK sufficiently prevents IFN-I activation, suggesting mtdsRNA release from BAX/BAK pore [170]. Importantly, evidences have been reported that mutations of PNTP1 cause accumulation of mtdsRNA in fibroblasts represented with novel type-I interferonopathy [171]. These studies highlighted a central role of mtdsRNA homeostasis and its function in inducing immune response with pathological consequences.

## Lifestyle preventive strategies for mtDNA mutation-induced tumorigenesis

**Figure 9.**
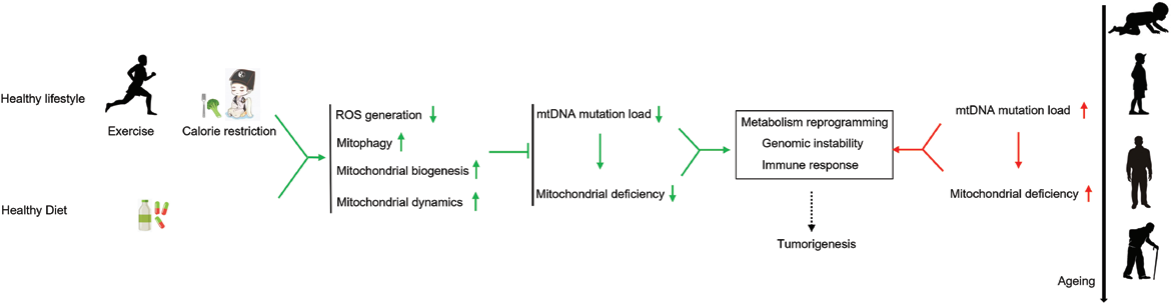
Schematic representation of healthy lifestyle in decreasing mtDNA mutations. Mitochondrial functional decline, as a result of mtDNA mutations accumulated during aging, can lead to metabolic reprogramming, genomic instability and immune response, contributing to tumorigenesis (red part to the right). Exercise, calories restriction and functional food could help reduce mtDNA mutation load and maintain mitochondrial function by decreasing ROS generation, enhancing mitophagy, mitochondrial biogenesis, and mitochondrial dynamics, which could decrease the risk of tumorigenesis (green part to the left).

Human life expectancy continues to increase in the next few decades, with 1 out of every 5 people expected to be more than 60 years old by 2050, and the life expectancy is even higher in certain countries [172]. It is important to maintain health in the elderly population in order to ensure independent lifestyle and reduce medical burden. As we discussed in the above sections, more accumulated mtDNA mutations will likely accompany with extended lifespan, which may, if our arguments are correct, result in an increase in cancer incidence. It is important to take strategies to slow down mtDNA mutation accumulation. Here we summarize some healthy lifestyles that could be helpful in managing mtDNA mutations (Fig. 9).

(1) Keep a healthy lifestyle such as calorie restriction and exercise. Lifestyle has been shown to play a pivotal role in cancer prevention [173]. Many studies have shown that intake of excess calories could lead to extra ROS production because excess calories will reduce respiration rate, and free electrons will leak from complex I and III to react with O_2_ to form super oxidants [2]. Calorie restriction can help reduce ROS production. Furthermore, it has been shown that calorie restriction could also lead to mitophagy, a process that can directly remove damaged mitochondria [174].

Low mitochondrial abundance and mitochondrial impairment were observed in sedentary elderly when compared to young or age-matched active elderly [175, 176]. Sedentary lifestyle will accelerate aging, mtDNA mutation accumulation and cancer susceptibility. Exercise could increase cytochrome c concentration and improve activities of key mitochondrial transcriptional factors such as PGC-1α, promoting mitochondrial biogenesis [177]. Meanwhile, exercise could enhance mitochondrial dynamics and increase respiratory supercomplex formation, which is important for preventing tumorigenesis [178]. It has also been shown that exercise can induce mitophagy at the cellular level [179].

(2) Intake of functional food components that can increase mitochondrial biogenesis and mitophagy. During aging, a significant reduction of mitochondrial turnover has been observed due to lower biogenesis and less effective mitophagy, further inducing the accumulation of mtDNA mutation [180]. Modulation of mitochondrial biogenesis and mitophagy could be a strategy to relieve and prevent aging-related tumorigenesis before mtDNA mutations reach pathogenic level. Many functional food components have been shown to play important roles in mitochondrial biogenesis and mitophagy regulation [52], such as resveratrol [181], spermadine [182] and urolithin A [68]. Studying these functional food components and understanding the underlying mechanisms for their function could provide a promising and effective strategy to maintain mtDNA integrity. Although forced mitophagy has been shown as a way to reduce the mtDNA heteroplasmy, it is non-selective to mutated mitochondria and the level can hardly be maintained [183].

## Conclusions and prospects

Even though most mtDNA heteroplasmic mutations are functionally recessive at the beginning, given enough time during aging, each new mtDNA mutation has certain probability to clonally expand to reach to pathogenic level in single-cells. Such cells will increase in numbers during aging, and mitochondrial functions eventually will be compromised at tissue and/or organ level. Tumorigenesis itself is a gradual and complex biological process in which mtDNA mutation may drive multiple effects, not merely by inducing OXPHOS deficiency, nuclear genome instability, or immune response, but through interactions among different pathways. Furthermore, many studies suggested that mitochondrial functions and OXPHOS can be essential for maintenance of tumor growth [184, 185], indicating the necessity to discuss the role of mtDNA mutations in tumor origin and tumor growth separately.

The ‘multi-hit’ theory in cancer biology has been popular in the past decades, and emerging evidences as collected in this essay indicate that accumulated mtDNA mutations may increase the likelihood to “multi-hit” in cancer development. In this context, it will be important for the translational and clinical scientists to take mtDNA mutations and mitochondrial defects into consideration when designing clinical medicine and trials in cancer prevention and treatment. Although we are arguing the important role of mtDNA mutations in cancer origin, it should be noted that a clear and direct link between mtDNA mutations and tumorigenesis has not been established. Even conceptually straightforward, mitochondrial genetics has not been convincingly evaluated regarding pro-tumorigenic effect of mtDNA mutations, mostly due to technological limitation. Below are two outstanding research directions that the authors consider critically needed in this endeavor.

(a) Understand mtDNA mutation accumulation dynamics during aging. Accumulation of mutated mtDNA to pathological levels usually lasts for decades in humans. However, dynamics involved in this process is still unclear. Clonal expansion of mtDNA mutation occurs independently in different cells, manifesting profound heterogeneity, and the process is very dynamic. At present, most direct observation is overall mutation load in bulk tissues at a single time point. Thus, it is critical to be able to capture mtDNA mutation load in many single-cells at different time points. We may therefore have the opportunity to quantify distribution of cellular mtDNA mutations within an individual patient, allowing us to understand the patterns and mechanisms involved in mtDNA mutation accumulation.
(b) Develop methods to manipulate mitochondrial genome. It is important to elucidate functional consequences of specific mtDNA mutations. Currently, stable genetically engineered mouse or human cell model that harbors different types of mtDNA mutations are largely not available. Technical limitation of manipulating mitochondrial genome is the major hurdle. An efficient tool to generate cell lines or model organisms harboring specific mtDNA mutations is necessary to demonstrate whether the relationship between mtDNA mutations and tumorigenesis is causal or merely correlative. Direct delivery of exogenous mtDNA with designed mutations into mitochondria has been reported, but it can hardly be repeated by other independent laboratories [186].

Regarding direct mtDNA editing, unlike the convenience of CRISPR-Cas9 based nuclear gene-editing, it is difficult to transfer RNA into mitochondria, which greatly prevents its development [187]. MtDNA with double-strand breaks (DSBs) is rapidly degraded in mitochondria [188], which enables specific control of mtDNA copy number by zinc-finger nuclease (ZFN) [189] or transcription activator-like effector nuclease (TALEN) [190]. Based on these strategies, two studies utilized mitochondrially targeted zinc-finger nuclease (mtZFN) and mitochondrially targeted transcription activator-like effector (mitoTALEN) realized *in vivo* reduction of m.5024C > T and recovery of phenotype [191, 192]. However, neither of the above methods directly changes or introduces mutations.

Recently, Mok et al. [193] have screened a deaminase enzyme that can edit C into T in mtDNA, and the method has been applied to edit mtDNA in mouse post-mitotic tissue [194]. Very recently, an upgraded DdCBE system was developed, which has improved editing efficiency by using wider editing contexts [195]. With this advance, DdCBE is a potentially useful editing method to correct mtDNA mutations. DdCBE-based mtDNA gene-editing tools have been applied in zebrafish [196], mouse [194, 197], rat [198] and human [199, 200]. However, it remains a high off-target ratio in mtDNA due to the effect of target spacing regions, and the off-target editing effect in nuclear genome is also high [201]. A novel method was just developed that enables mtDNA manipulation with more versatility, a giant leap in the field if confirmed and applied in different disease contexts [202].

As a unique organelle of cellular metabolism and signal transduction, mitochondria form an important link between environment and aging, and age-related diseases. Comprehensive understanding of mitochondrial homeostasis, especially through mtDNA mutations, will advance our knowledge of how mitochondria are involved in the development of age-related diseases, and provide a potential target for prevention and treatment of diseases, including cancer.
